# Association of lipid profile with obesity among breast cancer survivors: a cross-sectional study

**DOI:** 10.1186/s12944-022-01674-2

**Published:** 2022-08-02

**Authors:** Akinkunmi Paul Okekunle, Ga-Eun Yie, Sihan Song, Zisun Kim, Hyun Jo Youn, Jihyoung Cho, Jun Won Min, Yoo Seok Kim, Jung Eun Lee

**Affiliations:** 1 Department of Food and Nutrition, College of Human Ecology, Seoul National University, 1 Gwanak-ro, Gwanak-gu, Seoul, 08826 Korea; 2 Research Institute of Human Ecology, Seoul National University, Seoul, 08826 Korea; 3 Department of Surgery, Soonchunhyang University Bucheon Hospital, Soonchunhyang University College of Medicine, Bucheon, 14584 Korea; 4 Department of Surgery, Jeonbuk National University Medical School, Jeonju, 54907 Korea; 5 Department of Surgery, Keimyung University School of Medicine, Daegu, 42601 Korea; 6 Department of Surgery, Dankook University Hospital, Dankook University College of Medicine, Cheonan, 31116 Korea; 7 Department of Surgery, Chosun University Hospital, Chosun University College of Medicine, Gwangju, 61453 Korea

**Keywords:** Breast cancer, Lipids, Obesity, Metabolomics, ^1^H proton NMR, Menopause

## Abstract

**Background:**

The role of lipid metabolism in obesity and cancer manifestations cannot be underestimated, but whether alterations in lipid metabolism can manipulate the vasculature to promote obesity among breast cancer (BC) survivors is yet to be clearly understood. This study quantified plasma lipid and particle sizes using high-throughput proton (^1^H) nuclear magnetic resonance (NMR) and tested their associations with obesity among breast cancer (BC) survivors.

**Methods:**

A total of 348 (225 premenopausal and 123 postmenopausal) BC survivors enrolled from five hospitals in Korea were included. We assessed thirty-four plasma lipid biomarkers using ^1^H NMR, and obesity status was defined as a body mass index (BMI) of 25 kg/m^2^ or greater. Generalized linear and logistic regression models were applied to estimate the least-square means of BMI (kg/m^2^) and odds ratio (OR)s of obesity, respectively, and the corresponding 95% confidence interval (CI)s across plasma lipid levels.

**Results:**

Mean (SD) values of BMI was 23.3 (3.2) kg/m^2^ and 90 (25.9%) had BMI of ≥ 25 kg/m^2^. BMI levels increased with increasing total triglycerides (TG), TG in lipoproteins and very-low-density lipoprotein (VLDL) subfractions. However, BMI levels decreased with increasing tertiles of high-density lipoprotein (HDL)-cholesterol (C) and HDL particle size (HDL-p). Similar associations were observed in the logistic regression models. The increasing and decreasing BMI trends with TG and HDL profiles respectively were predominantly limited to premenopausal BC survivors.

**Conclusions:**

Increasing levels of plasma total TG and TG in lipoproteins were associated with increasing levels of BMI among premenopausal BC survivors. High HDL-C levels and large HDL-p were inversely associated with obesity among premenopausal BC survivors. Due to the cross-sectional design of this study, longitudinal studies are necessary to examine the association between obesity and lipid profile among BC survivors.

**Supplementary Information:**

The online version contains supplementary material available at 10.1186/s12944-022-01674-2.

## Introduction

Obesity is a public health challenge with a significant impact on the health and wellbeing of populations worldwide [1]. It is a significant risk factor for cancers [2], and cancer-specific mortality [3], accounting for an estimated 2.4 million deaths and 70.7 million disability-life adjusted years worldwide in 2017 [4] with a compelling cost of care [5]. It has been linked with increased breast cancer (BC) risk among postmenopausal women [6–8] with poor BC-specific survival [1, 9] but was inversely related to BC risk among premenopausal women [6, 10]. Anomalies in lipid metabolism are a common phenomenon in obesity [11].

Lipid metabolism cannot be underestimated in biologically vital intra-cellular processes, which are critical for normal metabolism and signal transduction [12, 13]. Similarly, lipid transduction may be essential to BC carcinogenesis and survivorship [14, 15]. Several reports [13, 16, 17] have documented the role of lipid metabolism in carcinogenesis, but there are limited data on the dynamics of lipid metabolism in cancer survival and recurrence. Whether adiposity could trigger changes in the vasculature to promote abnormal lipid metabolism among breast cancer (BC) survivors is yet to be clearly understood.

Understanding the association of obesity with plasma lipids among BC survivors will assist researchers and clinicians with vital information to discriminate the role of lipid metabolism in BC survival and recurrence. Similarly, discerning the contribution(s) of obesity to lipids metabolism among BC survivors and if peradventure it can manipulate the vasculature to promote the recurrence of tumour microenvironment might be vital in understanding the pathophysiology of lipid metabolism, adiposity and BC carcinogenesis.

Even though obesity has been linked with BC carcinogenesis, adiposity-related alterations in lipid composition(s) among BC survivors are yet to be explicitly enumerated. Exploring the significance of obesity in disordered plasma lipids and their sub-fraction(s) among BC survivors might help discern metabolic distinctions relevant for improving the quality of life and care of BC survivors. Obesity increases the risk of postmenopausal BC but is inversely associated with the risk of premenopausal BC in epidemiologic studies [6]. The mechanism through which adiposity confers different risks across menopausal status remains unknown, and investigating lipid profiles according to obesity status might help understand the complex biological nature of this phenomenon. Therefore, this study quantified plasma lipids using high-throughput proton (^1^H) nuclear magnetic resonance (NMR) and tested if they were associated with obesity among pre-and post-menopausal BC survivors in Korea.

## Methods

### Study population and design

This study is a cross-sectional report among female BC survivors enrolled in five hospitals in Korea between 2012 and 2019 (blood collection from 2015 to 2019). The Institutional Review Boards (IRB) of each hospital approved this study, and all participants provided written informed consent before participating in the study. All procedures performed in this study followed the ethical standards of the IRBs, the 1964 Helsinki declaration and its later amendments or comparable ethical standards.

Participants were diagnosed with primary BC stage I-III according to the American Joint Committee on Cancer (AJCC) criteria [18] and underwent BC surgery at least six months before enrollment. Out of 1184 BC survivors enrolled in the study, 463 participants' plasma samples were analyzed for metabolomic analysis. We further excluded participants for the following reasons; diagnosis of other forms of cancers before study enrolment (*n* = 14), BC recurrence or metastasis before enrolment or diagnosis with AJCC stage 0 or missing (*n* = 32) or missing information on body mass index (BMI) (*n* = 69). As a result, a total of 348 BC survivors (of which 123 were postmenopausal BC survivors) were included in the final analysis of this cross-sectional study.

### Sociodemographic, lifestyle and history of diseases

Recruitment and data collection of socio-demographic and lifestyle factors, health-behaviour, quality of life, dietary intake and clinical characteristics of participants in this study has been reported elsewhere [19–23]. Participants provided information on socio-demographic factors, including age (in years), marital status, and level of education. The age of participants was reported in years. Marital status was categorized as ‘never married’ (i.e. never been in any form of marital relationship), ‘married/cohabiting’ (i.e. in a marital relationship or living with a partner in a consensual relationship) and ‘divorced/separated/widowed’ (i.e. not in any form of marital relationship or union as a result of divorce or separation or death). Highest education completed was classified as ‘middle school or less’, ‘high school’ and ‘college education and above’. Smoking status was classified as ‘never’ if the participant reported no cigarette use or ‘ever smoked’ where the participant reported past or current cigarette use. Current alcohol use was reported as the current alcohol drinking status of the participant, and family history of BC was defined as any self-reported history of BC or breast diseases in first- or second-degree relatives. Participants reported information or diagnosis of chronic disease(s), including high blood pressure, hyperlipidemia, diabetes mellitus and stroke. Furthermore, participants provided information on the type, frequency and duration of various exercises and total metabolic equivalent (MET)-hour/week was estimated by summing all MET-hour/week of different types of exercise reported according to the Compendium of Physical Activities [24].

### Anthropometric assessment

Participants provided information on current weight (in kg) and height (in cm) through a questionnaire at enrollment. Body mass index (BMI) was estimated as weight (kg) divided by the square of height (m^2^). In this study, obesity was defined as having a BMI ≥ 25 kg/m^2^ for populations in the Asia–Pacific region according to the World Health Organization (Western Pacific Regional Office), the International Association for the Study of Obesity, and the International Obesity task force [25].

### Measurement of Lipid profile using High-throughput proton (^1^H) nuclear magnetic resonance (NMR) Metabolomics

Participants voluntarily provided consent for blood samples drawn by trained personnel. Blood samples were processed, sub-aliquoted, transported on dry ice for storage at – 80^0^C and later shipped for metabonomic analyses. High-throughput ^1^H NMR metabolomics (Nightingale Health Ltd, Helsinki, Finland) was employed to determine the concentration of various plasma lipids and their subfractions. Details of the principle and method of determination have been reported elsewhere [26, 27]. The ^1^H NMR method simultaneously quantified different low-molecular metabolites, including lipids, amino acids, ketone bodies and gluconeogenesis-related metabolites in molar concentration units. Details of the experimentation, applications, lipid extraction, determination, quantification, sub-class quantification and normalization in the ^1^H NMR Metabolomics procedure have been described elsewhere [27–29]. This study quantified total lipid and particle concentrations in fourteen lipoprotein subclasses and various subclass-specific lipid concentrations as total cholesterol and all the low-density lipoprotein subclasses (Supplementary Table 1). Representative coefficients of variations (CV) over thousands of samples for the metabonomic analysis were low-density lipoprotein cholesterol – LDL-C (2.3%), high-density lipoprotein cholesterol – HDL-C (2.3%), total cholesterol – TC (2.1%) and triglycerides – TG (1.2%). CVs for other plasma lipids were typically below 5%.

### Statistical analysis

Characteristics of BC survivors were compared by obesity status (BMI ≥ 25 kg/m^2^) using the chi-square test and independent-sample t-test for discrete and continuous data, respectively. Using a generalized linear model, we estimated and compared the least-square (LS) means and 95% confidence interval (CIs) of BMI across tertiles of plasma lipid concentrations. Change(s) in LS means and 95% CIs were assessed when including variables in the final model of the multivariate adjustment. Model 1 was adjusted for age (years, continuous) and menopausal status before BC diagnosis (no, yes). In Model 2, model 1 was additionally adjusted for highest education completed (middle school or less, high school, college education and above), ever smoked (no, yes), current alcohol use (no, yes) and family history of breast cancer (no, yes). Model 3 was additionally adjusted for a history of high blood pressure, dyslipidaemia, diabetes mellitus and stroke (no, yes) and physical activity (continuous, MET-hours/week). Test for trend was carried out by assigning the median value of tertile distribution as a continuous variable in the models. Also, the odds ratio (OR)s and 95% CIs of having obesity by tertile distribution of plasma lipid concentrations were estimated using logistic regression adjusting for the same covariates in the generalized linear models. Bonferroni’s correction was conducted for all *P* values in regression models to avoid the inflation of type I error. All statistical analyses were conducted using SAS 9.4 (SAS Institute Inc., Cary, NC, USA) at a two-sided *P*-value < 0.05.

## Results

### Characteristics of BC survivors in the study

Characteristics of BC survivors by obesity status (BMI ≥ 25 kg/m^2^) are presented in Table 1. Age, age at BC diagnosis, menopausal status at BC diagnosis, marital status, education, smoking and alcohol use differed insignificantly according to obesity status among BC survivors. Physical activity levels were higher among BC survivors with a BMI of < 25 kg/m^2^ than those with a BMI of ≥ 25 kg/m^2^. Mean BMI was 27.4 ± 2.2 kg/m^2^ among BC survivors with BMI of ≥ 25 kg/m^2^ and 21.9 ± 1.9 kg/m^2^ among those with BMI of < 25 kg/m^2^.

**Table 1:** Characteristics of breast cancer survivors according to BMI of 25 kg/m^2^ or greater

	BMI ≥ 25 kg/m^2^		
Characteristics	No	Yes	*P*-value
N	258	90	
Age^a^ (years), mean (SD)	52.2 (8.4)	52.2 (8.1)	0.94
Age at BC diagnosis (years), mean (SD)	48.7 (8.2)	48.9 (8.2)	0.81
Menopausal status before BC diagnosis, n (%)	89 (34.5)	34 (37.8)	0.58
Marital status, n (%)			
Never married	13 (5.1)	4 (4.5)	0.89
Married/Cohabiting	206 (79.8)	74 (82.2)	
Divorced/Separated/Widowed	39 (15.1)	12 (13.3)	
Highest education completed, n (%)			
Middle School	58 (22.5)	28 (31.1)	0.08
High School	134 (51.9)	48 (53.3)	
College education and above	66 (25.6)	14 (15.6)	
Ever smoker, n (%)	21 (8.1)	10 (11.1)	0.39
Current alcohol drinker, n (%)	56 (21.7)	16 (17.7)	0.42
Family history of BC^b^, n (%)	175 (67.8)	59 (65.6)	0.69
Presence or history of chronic disease^c^, n (%)	35 (13.6)	17 (18.9)	0.22
Physical Activity (MET-hours/week), mean (SD)	34.2 (35.4)	25.8 (28.9)	0.03
Height (cm), mean (SD)	158.4 (5.1)	157.6 (7.1)	0.34
BMI (kg/m^2^), mean (SD)	21.9 (1.9)	27.4 (2.2)	< 0.001

Continuous data are presented as mean (SD) and compared using the t-test

Categorical data are presented as n (%) and compared using the chi-squared test

^a^age at recruitment

^b^Family history of BC included a history of benign breast disease

^c^Chronic diseases included high blood pressure, dyslipidaemia, diabetes mellitus and stroke

### LS means and 95% CIs of BMI across the distribution of plasma lipid concentrations

Multivariable-adjusted LS means and 95% CIs of BMI across tertile distribution of plasma lipids are presented (Table 2 and Supplementary Table 2). Overall, LS means of BMI (in kg/m^2^) decreased across increasing tertiles distribution of high-density lipoprotein (HDL) subfractions of cholesterol, cholesteryl esters and lipoprotein particle sizes. For example, LS means of BMI increased across increasing tertile of average diameter for high-density lipoprotein particles (HDL-p); 23.9 (23.4, 24.5), 23.5 (22.9, 24.0) and 22.5 (21.9, 23.1; *P* for trend < 0.001). However, LS means of BMI increased across increasing tertile of very-low-density lipoprotein (VLDL) in subfractions of phospholipids, free cholesterol, total lipids, lipoprotein particle and lipoprotein particle sizes. For example, LS means and 95% CI of BMI across tertile distribution of average diameter for very low-density lipoprotein particles (VLDL-p) were 22.7 (22.2, 23.3), 23.3 (22.7, 23.8), and 23.9 (23.3, 24.4; P for trend = 0.009). BMI levels trend increasingly with increasing total TG and subfractions. When we stratified the analysis by menopausal status before BC diagnosis, we found that the significant associations were only limited to premenopausal BC survivors. When a Bonferroni corrected *P*-value threshold was used, p for trends for total-TG, VLDL-TG and HDL-p remained significant.

**Table 2:** Multivariable adjusted least square (LS) means and 95% confidence interval (CI)s of BMI (kg/m^2^) according to the distribution of lipid profiles among all breast cancer survivors

	LS means^a^ and 95% CIs of BMI (kg/m^2^) by plasma lipid markers			
Lipid Profile	Tertile 1	Tertile 2	Tertile 3	*P*-trend
Cholesterol (*m*mol/L)				
Total-C	23.7 (23.1, 24.3)	23.2 (22.6, 23.8)	22.9 (22.4, 23.6)	0.05
non-HDL-C	23.4 (22.9, 24.0)	23.3 (22.8, 23.9)	23.1 (22.5, 23.8)	0.56
Remnant-C	23.3 (22.7, 23.9)	23.3 (22.7, 23.8)	23.4 (22.8, 23.9)	0.79
VLDL-C	22.8 (22.3, 23.4)	23.6 (22.9, 24.1)	23.5 (22.9, 24.1)	0.17
Clinical LDL-C	23.7 (23.1, 24.3)	23.3 (22.8, 23.9)	22.8 (22.3, 23.4)	0.05
LDL-C^b^	23.7 (23.1, 24.2)	23.2 (22.6, 23.8)	23.0 (22.4, 23.6)	0.19
HDL-C	23.5 (22.9, 24.1)	23.7 (23.1, 24.2)	22.7 (22.1, 23.3)	0.03
Triglycerides (*m*mol/L)				
Total-TG	22.8 (22.2, 23.3)	22.9 (22.4, 23.5)	24.2 (23.6, 24.8)	< 0.001^§^
VLDL-TG	22.6 (22.1, 23.2)	23.1 (22.5, 23.7)	24.2 (23.6, 24.7)	< 0.001^§^
LDL-TG	22.9 (22.3, 23.5)	23.1 (22.5, 23.7)	23.9 (23.3, 24.4)	0.009
HDL-TG	22.9 (22.4, 23.6)	23.2 (22.7, 23.8)	23.7 (23.1,24.3)	0.008
Phospholipids (*m*mol/L)				
Total-PL	23.5 (22.9, 24.0)	23.2 (22.6, 23.7)	23.3 (22.7, 23.9)	0.45
VLDL-PL	22.8 (22.2, 23.3)	23.3 (22.7, 23.9)	23.8 (23.2, 24.4)	0.009
LDL-PL	23.7 (23.1, 24.2)	23.2 (22.6, 23.7)	23.1 (22.5, 23.6)	0.17
HDL-PL	23.4 (22.9, 24.0)	23.5 (22.9, 24.0)	22.9 (22.3, 23.5)	0.62
Cholesteryl esters (*m*mol/L)				
Total-CE	23.7 (23.1, 24.2)	23.4 (22.8, 23.9)	22.9 (22.3, 23.5)	0.07
VLDL-CE	22.9 (22.4, 23.5)	23.5 (22.9, 24.1)	23.4 (22.8, 23.9)	0.34
LDL-CE	23.5 (22.9, 24.1)	23.5 (22.9, 24.1)	22.9 (22.3, 23.5)	0.35
HDL-CE	23.7 (23.1, 24.2)	23.6 (23.0, 24.2)	22.7 (22.1, 23.2)	0.01
Free Cholesterol (*m*mol/L)				
Total-FC	23.2 (22.6, 23.8)	23.7 (23.1, 24.2)	22.9 (22.4, 23.6)	0.74
VLDL-FC	22.8 (22.3, 23.4)	23.3 (23.1, 24.3)	23.7 (23.1, 24.3)	0.04
LDL-FC	23.9 (23.3, 24.5)	23.2 (22.7, 23.8)	22.8 (22.2, 23.3)	0.005
HDL-FC	23.5 (22.9, 24.1)	23.3 (22.7, 23.9)	23.0 (22.5, 23.6)	0.13
Total Lipids (*m*mol/L)				
Total-L	23.4 (22.9, 24.1)	22.9 (22.4, 23.5)	23.5 (22.9, 24.0)	0.83
VLDL-L	22.9 (22.3, 23.4)	23.0 (22.5, 23.6)	24.0 (23.4, 24.6)	0.002
LDL-L	23.5 (22.9, 24.1)	23.4 (22.8, 23.9)	23.0 (22.5, 23.6)	0.34
HDL-L	23.4 (22.8, 24.0)	23.6 (23.0, 24.2)	22.9 (22.4, 23.5)	0.19
Lipoprotein particle (*m*mol/L)				
Total-LP	23.2 (22.7, 23.8)	23.7 (23.2, 24.3)	22.9 (22.3, 23.5)	0.39
VLDL-LP	22.9 (22.4, 23.6)	23.1 (22.5, 23.6)	23.9 (23.3, 24.4)	0.01
LDL-LP	23.3 (22.7, 23.9)	23.2 (22.7, 23.80	23.4 (22.8, 23.9)	0.89
HDL-LP	23.3 (22.7, 23.8)	23.6 (23.1, 24.2)	22.9 (22.4, 23.6)	0.27
Lipoprotein particle sizes (*n*m)				
VLDL-p	22.7 (22.2, 23.3)	23.3 (22.7, 23.8)	23.9 (23.3, 24.4)	0.009
LDL-p	23.7 (23.2, 24.3)	23.3 (22.7, 23.8)	22.9 (22.3, 23.5)	0.09
HDL-p	23.9 (23.4, 24.5)	23.5 (22.9, 24.0)	22.5 (21.9, 23.1)	< 0.001^§^

^a^Models were adjusted for age (years, continuous), menopausal status at BC diagnosis (pre-menopause, post-menopause), highest education completed (middle school, high school, college education and above), ever smoked (no, yes), current alcohol use (no, yes), family history of breast cancer (no, yes), history of chronic disease (no, yes) and physical activity (MET-hours/week, continuous)

^b^LDL-C was estimated from Friedewald’s equation. Clinical LDL-C and LDL-C are the same biomarker but refer to different definition methods [30]

^§^*P*-trend was signifincant at Bonferroni corrected *P*-value = 0.00147

### Odds ratio and 95% confidence interval of obesity status (BMI ≥ 25 kg/m^2^) distribution of plasma lipid concentrations

Multivariable-adjusted ORs and 95% CIs of obesity status across tertile distribution of plasma lipids among all BC survivors are presented in Table 3. The odds of obesity increased with increasing tertiles of total-TG and its subfractions. ORs and 95% CIs of obesity status in the third tertile of plasma lipid concentrations (using the first tertile as reference) were 1.95 (1.06, 3.59) for total TG, 2.21 (1.17, 4.16) for triglycerides in very-low-density lipoprotein (VLDL-TG), and 1.36 (1.75, 2.46) for triglycerides in low-density lipoprotein (LDL-TG). The odds of obesity decreased with increasing tertiles of HDL-C, cholesteryl esters in high-density lipoprotein (HDL-CE), and HDL-p; ORs (95% CIs) comparing the third tertile with the first tertile were 0.46 (0.24, 0.88) for HDL-C, 0.45 (0.23, 0.86) for HDL-CE, and 0.34 (0.18, 0.65) for HDL-p. When a Bonferroni corrected *P*-value threshold was used, *P* for trends for total-TG, HDL-CE, VLDL-L and HDL-p remained significant.

**Table 3:** Multivariable adjusted odds ratio (OR)s and 95% confidence interval (CI)s of BMI of 25 kg/m^2^ or greater according to the distribution of lipid profiles among all breast cancer survivors

	ORs^a^ and 95% CIs of BMI of 25 kg/m^2^ or greater by plasma lipid markers			
Lipid Profile	Tertile 1	Tertile 2	Tertile 3	*P*-trend
Cholesterol (*m*mol/L)				
Total-C	1.00	1.07 (0.59, 1.95)	0.74 (0.39, 1.39)	0.25
non-HDL-C	1.00	1.10 (0.60, 2.03)	0.99 (0.54, 1.83)	0.91
Remnant-C	1.00	1.28 (0.69, 2.38)	1.29 (0.69, 2.38)	0.36
VLDL-C	1.00	1.88 (0.99, 3.56)	1.86 (0.99, 3.50)	0.10
Clinical LDL-C	1.00	0.80 (0.44, 1.44)	0.67 (0.36, 1.23)	0.33
LDL-C^b^	1.00	0.84 (0.47, 1.52)	0.67 (0.36, 1.25)	0.27
HDL-C	1.00	0.89 (0.50, 1.59)	0.46 (0.24, 0.88)	0.01
Triglycerides (*m*mol/L)				
Total-TG	1.00	1.18 (0.62, 2.25)	1.95 (1.06, 3.59)	< 0.001^§^
VLDL-TG	1.00	1.63 (0.85, 3.13)	2.21 (1.17, 4.16)	0.02
LDL-TG	1.00	0.81 (0.43, 1.52)	1.36 (1.75, 2.46)	0.31
HDL-TG	1.00	1.01 (0.54, 1.91)	1.40 (0.77, 2.56)	0.09
Phospholipids (*m*mol/L)				
Total-PL	1.00	0.97 (0.53, 1.76)	0.87 (0.47, 1.61)	0.57
VLDL-PL	1.00	1.49 (0.78, 2.82)	1.98 (1.06, 3.68)	0.02
LDL-PL	1.00	0.82 (0.45, 1.48)	0.66 (0.36, 1.23)	0.20
HDL-PL	1.00	1.09 (0.61, 1.98)	0.65 (0.34, 1.23)	0.36
Cholesteryl esters (*m*mol/L)				
Total-CE	1.00	1.09 (0.60, 1.97)	0.70 (0.37, 1.33)	0.32
VLDL-CE	1.00	1.50 (0.80, 2.81)	1.61 (0.87, 2.98)	0.23
LDL-CE	1.00	0.94 (0.53, 1.70)	0.56 (0.30, 1.07)	0.52
HDL-CE	1.00	0.93 (0.52, 1.65)	0.45 (0.23, 0.86)	< 0.001^§^
Free Cholesterol (*m*mol/L)				
Total-FC	1.00	1.77 (0.97, 3.24)	0.98 (0.52, 1.87)	0.91
VLDL-FC	1.00	1.76 (0.93, 3.33)	2.02 (1.08, 3.80)	0.03
LDL-FC	1.00	0.63 (0.35, 1.13)	0.52 (0.28, 0.98)	0.05
HDL-FC	1.00	0.87 (0.48, 1.57)	0.64 (0.34, 1.19)	0.14
Total Lipids (*m*mol/L)				
Total-L	1.00	0.95 (0.51, 1.75)	1.16 (0.62, 2.14)	0.81
VLDL-L	1.00	1.23 (0.64, 2.35)	2.20 (1.19, 4.06)	< 0.001^§^
LDL-L	1.00	1.02 (0.57, 1.86)	0.78 (0.42, 1.46)	0.51
HDL-L	1.00	1.15 (0.63, 2.09)	0.62 (0.33, 1.18)	0.12
Lipoprotein particle (*m*mol/L)				
Total-LP	1.00	1.13 (0.62, 2.05)	0.70 (0.37, 1.31)	0.31
VLDL-LP	1.00	1.36 (0.72, 2.58)	1.99 (1.07, 3.70)	0.04
LDL-LP	1.00	0.95 (0.51, 1.76)	1.14 (0.63, 2.07)	0.75
HDL-LP	1.00	1.14 (0.63, 2.06)	0.71 (0.38, 1.33)	0.22
Lipoprotein particle sizes (*n*m)				
VLDL-p	1.00	1.72 (0.91, 3.28)	2.10 (1.12, 3.93)	0.03
LDL-p	1.00	0.56 (0.31, 1.03)	0.53 (0.29, 0.99)	0.02
HDL-p	1.00	0.65 (0.36, 1.16)	0.34 (0.18, 0.65)	< 0.001^§^

^a^Models were adjusted for age (years, continuous), menopausal status at BC diagnosis (pre-menopause, post-menopause), highest education completed (middle school, high school, college education and above), ever smoked (no, yes), current alcohol use (no, yes), family history of breast cancer (no, yes), history of chronic disease (no, yes) and physical activity (MET-hours/week, continuous)

^b^LDL-C was estimated from Friedewald’s equation. Clinical LDL-C and LDL-C are the same biomarker but refer to different definition methods [30]

^§^*P*-trend was signifincant at Bonferroni corrected *P*-value = 0.00147

Furthermore, the analysis was stratified by menopausal status prior to BC diagnosis (Tables 4 and 5), and the OR (95% CI) for odds of obesity across tertiles of plasma lipid concentrations among premenopausal BC survivors trended similarly as in the overall results in the following plasma lipids; total TG, VLDL-TG, phospholipids in very-low-density lipoprotein (VLDL-PL), free cholesterol in very-low-density lipoprotein (VLDL-FC), total lipids in very-low-density lipoprotein (VDL-L), very low-density lipoprotein particles (VLDL-LP), VLDL-p. HDL-CE and HDL-p. However, there was no statistically significant association between plasma lipid concentrations and obesity among postmenopausal BC survivors.

**Table 4:** Multivariable adjusted odds ratio (OR)s and 95% confidence interval (CI)s of BMI of 25 kg/m^2^ or greater according to the distribution of lipid profiles among premenopausal breast cancer survivors

	ORs^a^ and 95% CIs of BMI of 25 kg/m^2^ or greater by plasma lipid markers			
Lipid Profile	Tertile 1	Tertile 2	Tertile 3	*P*-trend
Cholesterol (*m*mol/L)				
Total-C	1.00	1.91 (0.87, 4.20)	0.90 (0.40, 2.02)	0.61
non-HDL-C	1.00	1.82 (0.83, 3.99)	1.35 (0.62, 2.96)	0.54
Remnant-C	1.00	2.10 (0.94, 4.71)	1.68 (0.76, 3.71)	0.26
VLDL-C	1.00	3.04 (1.34, 6.94)	2.43 (1.05, 5.60)	0.08
Clinical LDL-C	1.00	1.32 (0.62, 2.81)	0.86 (0.38, 1.91)	0.80
LDL-C^b^	1.00	1.54 (0.72, 3.32)	0.95 (0.43, 2.13)	0.91
HDL-C	1.00	0.99 (0.47, 2.07)	0.43 (0.19, 0.96)	0.04
Triglycerides (*m*mol/L)				
Total-TG	1.00	1.99 (0.88, 4.50)	2.74 (1.23, 6.10)	0.003
VLDL-TG	1.00	3.03 (1.34, 6.84)	2.81 (1.23, 6.46)	0.03
LDL-TG	1.00	1.30 (0.58, 2.90)	2.16 (0.99, 4.70)	0.11
HDL-TG	1.00	1.04 (0.47, 2.31)	1.74 (0.80, 3.80)	0.09
Phospholipids (*m*mol/L)				
Total-PL	1.00	1.26 (0.58, 2.76)	0.97 (0.45, 2.09)	0.96
VLDL-PL	1.00	2.27 (1.00, 5.15)	2.86 (1.27, 6.43)	0.01
LDL-PL	1.00	1.41 (0.66, 3.03)	0.90 (0.40, 1.99)	0.78
HDL-PL	1.00	1.25 (0.58, 2.70)	0.63 (0.29 1.38)	0.54
Cholesteryl esters (*m*mol/L)				
Total-CE	1.00	2.27 (1.02, 5.03)	0.98 (0.43, 2.21)	0.80
VLDL-CE	1.00	2.49 (1.10, 5.62)	2.25 (0.99, 5.11)	0.09
LDL-CE	1.00	1.05 (0.49, 2.25)	0.58 (0.27, 1.26)	0.69
HDL-CE	1.00	1.16 (0.55, 2.43)	0.46 (0.20, 1.03)	0.04
Free Cholesterol (*m*mol/L)				
Total-FC	1.00	2.72 (1.24, 5.99)	1.18 (0.51, 2.68)	0.64
VLDL-FC	1.00	2.08 (0.93, 4.63)	2.50 (1.13, 5.56)	0.03
LDL-FC	1.00	1.20 (0.56, 2.56)	0.70 (0.31, 1.57)	0.41
HDL-FC	1.00	0.99 (0.46, 2.11)	0.59 (0.27, 1.29)	0.18
Total Lipids (*m*mol/L)				
Total-L	1.00	1.23 (0.56, 2.72)	1.33 (0.61, 2.91)	0.50
VLDL-L	1.00	1.77 (0.77, 4.07)	3.24 (1.45, 7.21)	0.002
LDL-L	1.00	2.40 (1.10, 5.26)	1.15 (0.51, 2.62)	0.72
HDL-L	1.00	1.59 (0.73, 3.45)	0.72 (0.31, 1.61)	0.28
Lipoprotein particle (*m*mol/L)				
Total-LP	1.00	1.01 (0.47, 2.17)	0.69 (0.32, 1.50)	0.67
VLDL-LP	1.00	2.03 (0.88, 4.65)	2.99 (1.33, 6.69)	0.02
LDL-LP	1.00	1.51 (0.69, 3.33)	1.50 (0.70, 3.24)	0.33
HDL-LP	1.00	1.04 (0.48, 2.25)	0.77 (0.36, 1.66)	0.44
Lipoprotein particle sizes (*n*m)				
VLDL-p	1.00	2.12 (0.93, 4.85)	3.16 (1.41, 7.03)	0.004
LDL-p	1.00	0.83 (0.40, 1.71)	0.53 (0.24, 1.18)	0.08
HDL-p	1.00	0.60 (0.28, 1.26)	0.28 (0.13, 0.64)	< 0.001^§^

^a^Models were adjusted for age (years, continuous), highest education completed (middle school, high school, college education and above), ever smoked (no, yes), current alcohol use (no, yes), family history of breast cancer (no, yes), history of chronic disease (no, yes) and physical activity (MET-hours/week, continuous)

^b^LDL-C was estimated from Friedewald’s equation. Clinical LDL-C and LDL-C are the same biomarker but refer to different definition methods [30]

^§^*P*-trend was signifincant at Bonferroni corrected *P*-value = 0.00147

**Table 5:** Multivariable adjusted odds ratio (OR)s and 95% confidence interval (CI)s of BMI of 25 kg/m^2^ or greater according to the distribution of lipid profiles among postmenopausal breast cancer survivors

	ORs^a^ and 95% CIs of BMI of 25 kg/m^2^ or greater by plasma lipid markers			
Lipid Profile	Tertile 1	Tertile 2	Tertile 3	*P*-trend
Cholesterol (*m*mol/L)				
Total-C	1.00	0.99 (0.98, 1.01)	0.72 (0.36, 1.41)	0.33
non-HDL-C	1.00	0.47 (0.15, 1.42)	0.63 (0.21, 1.89)	0.55
Remnant-C	1.00	0.62 (0.21, 1.84)	0.76 (0.26, 2.29)	0.86
VLDL-C	1.00	0.82 (0.26, 2.57)	0.90 (0.30, 2.73)	0.81
Clinical LDL-C	1.00	0.28 (0.09, 0.89)	0.52 (0.17, 1.60)	0.34
LDL-C^b^	1.00	0.43 (0.14, 1.26)	0.44 (0.14, 1.41)	0.20
HDL-C	1.00	0.96 (0.35, 2.60)	0.61 (0.19, 2.05)	0.21
Triglycerides (*m*mol/L)				
Total-TG	1.00	0.58 (0.10, 1.19)	0.76 (0.27, 2.18)	0.96
VLDL-TG	1.00	0.33 (0.09, 1.21)	0.80 (0.26, 2.49)	0.89
LDL-TG	1.00	0.37 (0.11, 1.23)	0.51 (0.18, 1.43)	0.43
HDL-TG	1.00	1.23 (0.35, 4.21)	0.79 (0.28, 2.25)	0.96
Phospholipids (*m*mol/L)				
Total-PL	1.00	0.76 (0.28, 2.10)	0.69 (0.22, 2.16)	0.31
VLDL-PL	1.00	0.48 (0.15, 1.56)	0.65 (0.21, 2.05)	0.73
LDL-PL	1.00	0.43 (0.15, 1.26)	0.44 (0.14, 1.41)	0.16
HDL-PL	1.00	0.94 (0.34, 2.59)	0.73 (0.22, 2.37)	0.44
Cholesteryl esters (*m*mol/L)				
Total-CE	1.00	0.53 (0.19, 1.48)	0.54 (0.16, 1.78)	0.32
VLDL-CE	1.00	0.69 (0.22, 2.14)	0.77 (0.26, 2.24)	0.54
LDL-CE	1.00	0.85 (0.31, 2.30)	0.57 (0.31, 2.30)	0.29
HDL-CE	1.00	0.80 (0.30, 2.13)	0.59 (0.17, 1.80)	0.18
Free Cholesterol (*m*mol/L)				
Total-FC	1.00	0.96 (0.34, 2.75)	0.79 (0.25, 2.51)	0.71
VLDL-FC	1.00	1.08 (0.33, 3.52)	1.08 (0.32, 3.55)	0.98
LDL-FC	1.00	0.25 (0.08, 0.78)	0.51 (0.17, 0.78)	0.16
HDL-FC	1.00	0.86 (0.28, 2.64)	0.86 (0.28, 2.64)	0.62
Total Lipids (*m*mol/L)				
Total-L	1.00	0.69 (0.24, 1.99)	0.85 (0.28, 2.54)	0.66
VLDL-L	1.00	0.46 (0.14, 1.50)	0.80 (0.26, 2.42)	0.98
LDL-L	1.00	0.31 (0.11, 0.95)	0.57 (0.18, 1.75)	0.30
HDL-L	1.00	0.81 (0.29, 2.24)	0.49 (0.15, 1.62)	0.24
Lipoprotein particle (*m*mol/L)				
Total-LP	1.00	1.59 (0.56, 4.52)	0.65 (0.20, 2.15)	0.35
VLDL-LP	1.00	0.71 (0.23, 2.15)	0.74 (0.24, 2.25)	0.73
LDL-LP	1.00	0.44 (0.14, 1.38)	0.65 (0.23, 1.88)	0.40
HDL-LP	1.00	1.40 (0.50, 3.87)	0.60 (0.18, 2.00)	0.41
Lipoprotein particle sizes (*n*m)				
VLDL-p	1.00	0.98 (0.32, 3.02)	0.68 (0.22, 2.15)	0.39
LDL-p	1.00	0.25 (0.08, 0.83)	0.53 (0.18, 1.56)	0.18
HDL-p	1.00	0.66 (0.23, 1.88)	0.46 (0.14, 1.55)	0.26

^a^Models were adjusted for age (years, continuous), highest education completed (middle school, high school, college education and above), ever smoked (no, yes), current alcohol use (no, yes), family history of breast cancer (no, yes), history of chronic disease (no, yes) and physical activity (MET-hours/week, continuous)

^b^LDL-C was estimated from Friedewald’s equation. Clinical LDL-C and LDL-C are the same biomarker but refer to different definition methods [30]

## Discussion

This study measured lipid profiles in plasma using the ^1^H NMR Metabolomics procedure and assessed their relationship with obesity among BC survivors in Korea. First, we observed an obesity-related increase in total TG and TG subfraction concentrations in VLDL among BC survivors. Second, we found decreased BMI trends were associated with increasing plasma HDL-CE and HDL-p. Third, of these associations, plasma HDL-p was inversely associated with obesity primarily among premenopausal BC survivors only after Bonferroni correction.

In tandem with our observation, some epidemiologic studies have demonstrated that the HDL-p (compared with other lipid biomarkers) is reliable for predicting populations at risk of cardiovascular diseases [31–33]. Similarly, lower HDL-p profiles have been reported among metabolically unhealthy subjects [34], subjects with coronary artery disease [35], impaired insulin sensitivity [36] and increased carotid media thickness [32]. Even in normal-weight subjects, structural alterations in HDL-p were associated with worsening metabolic health [34]. Furthermore, low HDL-p has been a reliable indicator for hypertriglyceridaemia among abdominally obese subjects [33].

Different mechanisms [32, 34, 37, 38] explain the beneficial inverse association of obesity-related HDL-p alterations. First, a feasible explanation is a relationship between HDL particle subfractions-2b (HDL-2b) and fractional rate cholesterol esterification (FER-HDL). FER-HDL is a suitable measure for defining HDL potential to esterify free cholesterol [37] and positively correlates with BMI [38]. When free VLDL/LDL are unesterified, they are traditionally stored as excess fat in the fatty tissues [39]. However, FER-HDL inversely correlates with HDL-2b [38]. Second, the inverse association of HDL-p with obesity might be related to the ATP-binding cassette transporters-mediated release of large HDL particles to avert cellular lipid accretion in the hepatic cells and macrophages [40–42]. Third, HDL particle subfractions 2b have been reported to exert anti-inflammatory potential by negatively manipulating lymphocyte activation and exhibiting an inverse relationship with inflammation markers (such as; high-sensitivity C-reactive protein, interleukin-6) among metabolically unhealthy obese subjects [34].

Our study revealed that VLDL subfractions were elevated among premenopausal BC survivors with high BMI. Of particular importance, we observed that higher VLDL concentrations in lipoprotein particle size was associated with higher odds of obesity. This observation was common to findings from other studies where elevated VLDL subclasses were observed among obese adolescents [43, 44]. The significance of high LDL in tumour carcinogenesis has been reported [13, 16, 17]. The role of VLDL profile as a risk factor for atherosclerosis [45], myocardial infarction [46], how it manipulates ATP-binding cassette transporters [47, 48] and its oxidative stress-mediation capacity [49] to promote the anomalous accumulation of triglycerides [48] have been documented.

Furthermore, our study showed notable differences in the association of obesity with plasma lipids between pre-and post-menopausal BC survivors and could imply variances in the aetiology of plasma lipid metabolism between pre-and post-menopausal BC survivors. The significance of menopause in the association of plasma lipids with adiposity is still unclear. However, a plausible rationale might be age- and hormonal-related (due to menopause) functional abnormalities in lipid metabolism hypothesized in some reviews [50–53]. Post-menopausal estrogen deficiency has been linked to a constellation of adverse metabolic changes that include dysregulated lipid metabolism [52] and may further be responsible for alterations in the secretion of pancreatic enzymes vital in lipid metabolism [50]. For example, low paraoxonase-1 activity (a hydrolytic enzyme involved in various functions to prevent lipid oxidation [54]) has been reported among healthy postmenopausal women compared to premenopausal women [55].

On the contrary, a clinical trial has reported a null association between cholesterol-based particles, non-esterified fatty acids and measures of adiposity among healthy postmenopausal women [56]. Limited evidence supports that these alterations are exclusively related to ageing and hormonal changes. More importantly, our study presents evidence alluding to the significance of obesity in the alteration of low-molecular-weight subfractions of plasma lipid profiles among BC survivors that might be pertinent for effective decision-making in improving the quality of life and care of BC survivors. The importance of early identification of populations at risk of alterations in lipid metabolism that could predispose to cardiovascular abnormalities cannot be undervalued in improving the quality of life and care of BC survivors. More studies are necessary to clarify these associations. Anthropometric measure at enrollment was self-reported in this study. However, weight at breast cancer diagnosis was measured using a standard instrument and strongly correlated with the self‐reported weight at study enrollment (Pearson’s *r* = 0.93, *p* < 0.001), suggesting the self-reported weight data were reliable. BMI is a reasonable surrogate for screening body fatness, but it could be subject to misclassification in the overall body composition assessment. We are constrained by limited data on the percentage body fat and free fat mass. This study cannot infer causal associations between lipid profile and obesity due to the cross-sectional design. The generalizability of our findings across a multi-ethnic population is limited, given that our subjects were exclusively Koreans. Longitudinal studies across diverse ethnic backgrounds might clarify the causal associations of plasma lipid biomarkers with obesity among BC survivors.

## Conclusion

In this study, higher TG and VLDL subfractions of plasma lipids were associated with higher odds of obesity, but higher plasma HDL-p was inversely related to the odds of obesity predominantly among premenopausal BC survivors.

## Supplementary Information

**Additional file 1:** **Supplementary Table 1.** A list of lipid biomarkers assayed. **Supplementary Table 2.** Multivariable adjusted least square (LS) means and 95% confidence interval (CI)s of BMI (kg/m2) according to the distribution of lipid profiles among all breast cancer survivors.

## Data Availability

The data for this study cannot be made publicly available because it is an ongoing study, and during the signing of consent, the participants were not informed that their information would be stored in a publicly accessible database. However, other researchers may collaborate with the study team upon request. Requests to access the data may be sent to the corresponding author.

## Abbreviations

AJCC: American Joint Committee on Cancer
BC: Breast cancer
BMI: Body mass index
CV: Coefficients of variations
CI: Confidence interval
^1^H: High-throughput proton
LS: Least-square
MET: Metabolic equivalent task
NMR: Nuclear magnetic resonance
OR: Odds ratio
TC: Total cholesterol
HDL-C: High-density lipoprotein cholesterol
non-HDL-C: Total cholesterol minus HDL-C
Remnant-C: Remnant cholesterol (non-HDL, non-LDL -cholesterol)
VLDL-C: Very low-density lipoprotein cholesterol
Clinical LDL-C: Clinical low-density lipoprotein cholesterol
LDL-C: Low density lipoprotein cholesterol – derived from the Friedewald’s equation for estimating LDL (56)
TG: Total triglycerides
VLDL-TG: Triglycerides in very-low-density lipoprotein
LDL-TG: Triglycerides in low-density lipoprotein
HDL-TG: Triglycerides in high-density lipoprotein
Total-PL: Total phospholipids in lipoprotein particles
VLDL-PL: Phospholipids in very-low-density lipoprotein
LDL-PL: Phospholipids in low-density lipoprotein
HDL-PL: Phospholipids in high-density lipoprotein
Total-CE: Total esterified cholesterol
VLDL-CE: Cholesteryl esters in very low-density lipoprotein
LDL-CE: Cholesteryl esters in low-density lipoprotein
HDL-CE: Cholesteryl esters in high-density lipoprotein
Total-FC: Total free cholesterol
VLDL-FC: Free cholesterol in very-low-density lipoprotein
LDL-FC: Free cholesterol in low-density lipoprotein
HDL-FC: Free cholesterol in high-density lipoprotein
Total-L: Total lipids in lipoprotein particles
VLDL-L: Total lipids in very-low-density lipoprotein
LDL-L: Total lipids in low-density lipoprotein
HDL-L: Total lipids in high-density lipoprotein
Total-LP: Total concentration of lipoprotein particles
VLDL-LP: Very low-density lipoprotein particles
LDL-LP: Low-density lipoprotein particles
HDL-LP: High-density lipoprotein particles
VLDL-p: Average diameter for very low-density lipoprotein particles
LDL-p: Average diameter for low-density lipoprotein particles
HDL-p: Average diameter for high-density lipoprotein particles
