# Supplementary material for: Association of lipid profile with obesity among breast cancer survivors: a cross-sectional study
Source: Lipids Health Dis. 2022 Aug 2;21:66. doi: 10.1186/s12944-022-01674-2 (PMC9344652; doi:10.1186/s12944-022-01674-2)
Supplement: Supplementary file 1 — Additional file 1: Supplementary Table 1. A list of lipid biomarkers assayed. Supplementary Table 2. Multivariable adjusted least square (LS) means and 95% confidence interval (CI)s of BMI (kg/m2) according to the distribution of lipid profiles among all breast cancer survivors. [file 12944_2022_1674_MOESM1_ESM.docx]

Supplementary Table 1: A list of lipid biomarkers assayed

| Lipid biomarkers | Abbreviations |
| --- | --- |
| Cholesterol (*m*mol/L) |  |
| Total cholesterol | Total-C |
| Non-high-density lipoprotein cholesterol | non-HDL-C |
| Remnant cholesterol (non-high-density lipoprotein cholesterol and non-low-density lipoprotein cholesterol) | Remnant-C |
| Very low-density lipoprotein cholesterol | VLDL-C |
| Clinical low-density lipoprotein cholesterol | Clinical LDL-C |
| Low-density lipoprotein cholesterol | LDL-C |
| High-density lipoprotein cholesterol | HDL-C |
| Triglycerides (*m*mol/L) |  |
| Total triglycerides | Total-TG |
| Triglycerides in very-low-density lipoprotein | VLDL-TG |
| Triglycerides in low-density lipoprotein | LDL-TG |
| Triglycerides in high-density lipoprotein | HDL-TG |
| Phospholipids (*m*mol/L) |  |
| Total phospholipids in lipoprotein particles | Total-PL |
| Phospholipids in very-low-density lipoprotein | VLDL-PL |
| Phospholipids in low-density lipoprotein | LDL-PL |
| Phospholipids in high-density lipoprotein | HDL-PL |
| Cholesteryl esters (*m*mol/L) |  |
| Total esterified cholesterol | Total-CE |
| Cholesteryl esters in very-low-density lipoprotein | VLDL-CE |
| Cholesteryl esters in low-density lipoprotein | LDL-CE |
| Cholesteryl esters in high-density lipoprotein | HDL-CE |
| Free Cholesterol (*m*mol/L) |  |
| Total free cholesterol | Total-FC |
| Free cholesterol in very-low-density lipoprotein | VLDL-FC |
| Free cholesterol in low-density lipoprotein | LDL-FC |
| Free cholesterol in high-density lipoprotein | HDL-FC |
| Total Lipids (*m*mol/L) |  |
| Total lipids in lipoprotein particles | Total-L |
| Total lipids in very-low-density lipoprotein | VLDL-L |
| Total lipids in low-density lipoprotein | LDL-L |
| Total lipids in high-density lipoprotein | HDL-L |
| Lipoprotein particle (*m*mol/L) |  |
| Total lipoprotein particles | Total-LP |
| Very low-density lipoprotein particles | VLDL-LP |
| Low-density lipoprotein particles | LDL-LP |
| High-density lipoprotein particles | HDL-LP |
| Lipoprotein particle sizes (*n*m) |  |
| Average diameter of very-low-density lipoprotein particle sizes | VLDL-p |
| Average diameter of low-density lipoprotein particle sizes | LDL-p |
| Average diameter of high-density lipoprotein particle sizes | HDL-p |

Supplementary Table 2: Multivariable adjusted least square (LS) means and 95% confidence interval (CI)s of BMI (kg/m^2^) according to the distribution of lipid profiles among all breast cancer survivors

|  | LS means and 95% CIs of BMI (kg/m^2^) by plasma lipid markers | | | |
| --- | --- | --- | --- | --- |
| Lipid Profile | Tertile 1 | Tertile 2 | Tertile 3 | *P*-trend |
| Cholesterol (*m*mol/L) |  |  |  |  |
| Total-C |  |  |  |  |
| Model 1 | 23.7 (23.1, 23.3) | 23.3 (22.7, 23.9) | 22.9 (22.4, 23.5) | 0.05 |
| Model 2 | 23.7 (23.1, 24.3) | 23.2 (22.6, 23.8) | 22.9 (22.4, 23.5) | 0.04 |
| non-HDL-C |  |  |  |  |
| Model 1 | 23.5 (22.9. 24.1) | 23.3 (22.7, 23.9) | 23.1 (22.5, 23.7) | 0.53 |
| Model 2 | 23.5 (22.9, 24.0) | 23.3 (22.7, 23.9) | 23.1 (22.5, 23.7) | 0.54 |
| Remnant-C |  |  |  |  |
| Model 1 | 23.3 (22.7, 23.9) | 23.2 (22.6, 23.8) | 23.4 (22.8, 23.9) | 0.70 |
| Model 2 | 23.4 (22.8, 23.9) | 23.2 (22.6, 23.8) | 23.4 (22.8, 23.9) | 0.84 |
| VLDL-C |  |  |  |  |
| Model 1 | 22.8 (22.2, 23.4) | 23.6 (22.9, 24.1) | 23.6 (22.9, 24.1) | 0.11 |
| Model 2 | 22.8 (22.3, 23.4) | 23.5 (22.9, 24.1) | 23.5 (22.9, 24.1) | 0.17 |
| Clinical LDL-C |  |  |  |  |
| Model 1 | 23.7 (23.2, 24.3) | 23.4 (22.8, 23.9) | 22.8 (22.2, 23.4) | 0.04 |
| Model 2 | 23.7 (23.2, 24.3) | 23.4 (22.8, 23.9) | 22.8 (22.2, 23.3) | 0.03 |
| LDL-C^*^ |  |  |  |  |
| Model 1 | 23.6 (23.0, 24.1) | 23.3 (22.7, 23.9) | 22.9 (22.4, 23.5) | 0.15 |
| Model 2 | 23.7 (23.0, 24.2) | 23.3 (22.7, 23.9) | 23.0 (22.4, 23.5) | 0.15 |
| HDL-C |  |  |  |  |
| Model 1 | 23.5 (22.9, 24.1) | 23.7 (23.1, 24.1) | 22.7 (22.1, 23.3) | 0.03 |
| Model 2 | 23.4 (22.9, 24.0) | 23.7 (23.2, 24.3) | 22.7 (21.1, 23.3) | 0.04 |
| Triglycerides (*m*mol/L) |  |  |  |  |
| Total-TG |  |  |  |  |
| Model 1 | 22.7 (22.1, 23.2) | 22.9 (22.4, 23.5) | 24.3 (23.7, 24.8) | <0.001^§^ |
| Model 2 | 22.7 (22.2, 23.3) | 22.9 (22.4, 24.5) | 24.2 (23.7, 24.8) | <0.001^§^ |
| VLDL-TG |  |  |  |  |
| Model 1 | 22.5 (21.9, 23.1) | 23.2 (22.6, 23.7) | 24.2 (23.6, 24.8) | <0.001^§^ |
| Model 2 | 22.6 (22.0, 23.2) | 23.1 (22.6, 23.7) | 24.2 (23.6, 24.8) | <0.001^§^ |
| LDL-TG |  |  |  |  |
| Model 1 | 22.9 (22.3, 23.5) | 23.1 (22.5, 23.7) | 23.9 (23.4, 24.5) | 0.005 |
| Model 2 | 22.9 (22.3, 23.5) | 23.1 (22.5, 23.7) | 23.9 (23.3, 24.5) | 0.009 |
| HDL-TG |  |  |  |  |
| Model 1 | 22.9 (22.4, 23.5) | 23.2 (22.6, 23.7) | 23.8 (23.2, 24.4) | 0.003 |
| Model 2 | 22.9 (22.3, 23.5) | 23.2 (22.7, 23.8) | 23.7 (23.2, 24.3) | 0.004 |
| Phospholipids (*m*mol/L) |  |  |  |  |
| Total-PL |  |  |  |  |
| Model 1 | 23.4 (22.8, 23.9) | 23.2 (22.6, 23.8) | 23.3 (22.7, 23.9) | 0.63 |
| Model 2 | 23.4 (22.9, 24.0) | 23.2 (22.6, 23.8) | 23.3 (22.7, 23.9) | 0.53 |
| VLDL-PL |  |  |  |  |
| Model 1 | 22.7 (22.2, 23.3) | 23.3 (22.7, 23.9) | 23.8 (23.3, 24.4) | 0.005 |
| Model 2 | 22.8 (22.2, 23.3) | 23.3 (22.7, 23.9) | 23.8 (23.3, 24.4) | 0.008 |
| LDL-PL |  |  |  |  |
| Model 1 | 23.6 (23.1, 24.2) | 23.2 (22.7, 23.8) | 23.0 (22.5, 23.6) | 0.17 |
| Model 2 | 23.7 (23.1, 24.2) | 23.2 (22.6, 23.8) | 23.0 (22.4, 23.6) | 0.15 |
| HDL-PL |  |  |  |  |
| Model 1 | 23.4 (22.7, 23.9) | 23.6 (23.0, 24.2) | 22.9 (22.4, 23.5) | 0.66 |
| Model 2 | 23.3 (22.8, 23.9) | 23.6 (23.0, 24.2) | 22.9 (22.4, 23.6) | 0.81 |
| Cholesteryl esters (*m*mol/L) |  |  |  |  |
| Total-CE |  |  |  |  |
| Model 1 | 23.6 (23.0, 24.2) | 23.5 (22.9, 24.1) | 22.8 (22.2, 23.4) | 0.07 |
| Model 2 | 23.6 (23.1, 24.2) | 23.4 (22.8, 23.9) | 22.8 (22.3, 23.4) | 0.06 |
| VLDL-CE |  |  |  |  |
| Model 1 | 22.9 (22.4, 23.5) | 23.5 (22.9, 24.1) | 23.5 (22.9, 24.0) | 0.27 |
| Model 2 | 22.9 (22.4, 23.6) | 23.5 (22.9, 24.1) | 23.4 (22.9, 24.0) | 0.33 |
| LDL-CE |  |  |  |  |
| Model 1 | 23.4 (22.9, 24.0) | 23.5 (22.9, 24.1) | 22.9 (22.3, 23.4) | 0.32 |
| Model 2 | 23.4 (22.9, 24.1) | 23.5 (22.9, 24.1) | 22.9 (22.3, 23.5) | 0.29 |
| HDL-CE |  |  |  |  |
| Model 1 | 23.6 (23.0, 24.2) | 23.7 (23.2, 24.2) | 22.6 (22.0, 23.1) | 0.01 |
| Model 2 | 23.6 (23.0, 24.2) | 23.6 (23.1, 24.2) | 22.6 (22.1, 23.2) | 0.01 |
| Free Cholesterol (*m*mol/L) |  |  |  |  |
| Total-FC |  |  |  |  |
| Model 1 | 23.2 (22.6, 23.8) | 23.7 (23.1, 24.2) | 22.9 (22.4, 23.6) | 0.74 |
| Model 2 | 23.2 (22.6, 23.8) | 23.7 (23.1, 24.2) | 22.9 (22.4, 23.5) | 0.71 |
| VLDL-FC |  |  |  |  |
| Model 1 | 22.8 (22.2, 23.4) | 23.3 (22.8, 23.9) | 23.8 (23.2, 24.4) | 0.03 |
| Model 2 | 22.8 (22.2, 23.4) | 23.3 (22.8, 23.9) | 23.7 (23.1, 24.3) | 0.04 |
| LDL-FC |  |  |  |  |
| Model 1 | 23.9 (23.4, 24.5) | 23.2 (22.7, 23.8) | 22.7 (22.1, 23.3) | 0.002 |
| Model 2 | 23.9 (23.4, 24.5) | 23.3 (22.7, 23.9) | 22.7 (22.1, 23.3) | 0.002 |
| HDL-FC |  |  |  |  |
| Model 1 | 23.4 (22.9, 24.0) | 23.4 (22.8, 23.9) | 23.0 (22.4, 23.5) | 0.17 |
| Model 2 | 23.5 (22.9, 24.1) | 23.4 (22.8, 23.9) | 23.5 (22.9, 24.1) | 0.17 |
| Total Lipids (*m*mol/L) |  |  |  |  |
| Total-L |  |  |  |  |
| Model 1 | 23.4 (22.8, 23.9) | 22.9 (22.4, 23.9) | 23.5 (22.9, 24.1) | 0.86 |
| Model 2 | 23.4 (22.9, 24.0) | 22.9 (22.4, 23.5) | 23.5 (22.9, 24.0) | 0.91 |
| VLDL-L |  |  |  |  |
| Model 1 | 22.8 (22.2, 23.4) | 22.9 (22.4, 23.6) | 24.1 (23.5, 24.7) | <0.001^§^ |
| Model 2 | 22.8 (22.3, 23.4) | 23.0 (22.4, 23.6) | 24.1 (23.5, 24.6) | 0.001^§^ |
| LDL-L |  |  |  |  |
| Model 1 | 23.4 (22.9, 24.0) | 23.4 (22.8, 24.0) | 23.0 (22.5, 23.6) | 0.38 |
| Model 2 | 23.5 (22.9, 24.1) | 23.4 (22.8, 23.9) | 23.0 (22.4, 23.6) | 0.34 |
| HDL-L |  |  |  |  |
| Model 1 | 23.3 (22.7, 23.9) | 23.7 (23.1, 24.2) | 22.9 (22.4, 23.5) | 0.23 |
| Model 2 | 23.3 (22.7, 23.9) | 23.7 (23.1, 24.2) | 22.9 (22.4, 23.5) | 0.27 |
| Lipoprotein particle (*m*mol/L) |  |  |  |  |
| Total-LP |  |  |  |  |
| Model 1 | 23.2 (22.6, 23.8) | 23.7 (23.2, 24.3) | 22.9 (22.3, 23.5) | 0.43 |
| Model 2 | 23.2 (22.6, 23.8) | 23.8 (23.2, 24.4) | 22.9 (22.4, 23.5) | 0.55 |
| VLDL-LP |  |  |  |  |
| Model 1 | 22.9 (22.3, 23.5) | 23.1 (22.5, 23.6) | 23.9 (23.4, 24.5) | 0.007 |
| Model 2 | 22.9 (22.4, 23.5) | 23.1 (22.5, 23.6) | 23.9 (23.3, 24.5) | 0.01 |
| LDL-LP |  |  |  |  |
| Model 1 | 23.3 (22.8, 23.9) | 23.1 (22.5, 23.7) | 23.5 (22.9, 24.0) | 0.87 |
| Model 2 | 23.3 (22.8, 23.9) | 23.1 (22.6, 23.7) | 23.4 (22.8, 23.9) | 0.99 |
| HDL-LP |  |  |  |  |
| Model 1 | 23.2 (22.7, 23.8) | 23.7 (23.4, 24.2) | 22.9 (22.4, 23.6) | 0.32 |
| Model 2 | 23.2 (22.6, 23.8) | 23.7 (23.1, 24.3) | 23.0 (22.4, 23.6) | 0.44 |
| Lipoprotein particle sizes (*n*m) |  |  |  |  |
| VLDL-p |  |  |  |  |
| Model 1 | 22.7 (22.1, 23.3) | 23.3 (22.7, 23.8) | 23.9 (23.3, 24.5) | 0.005 |
| Model 2 | 22.7 (22.1, 23.3) | 23.3 (22.7, 23.8) | 23.9 (23.3, 24.5) | 0.006 |
| LDL-p |  |  |  |  |
| Model 1 | 23.7 (23.1, 24.3) | 23.4 (22.8, 23.9) | 22.9 (22.3, 23.4) | 0.11 |
| Model 2 | 23.7 (23.1, 24.3) | 23.3 (22.8, 23.9) | 22.8 (22.3, 23.4) | 0.10 |
| HDL-p |  |  |  |  |
| Model 1 | 23.9 (23.4, 24.5) | 23.5 (22.9, 24.1) | 22.4 (21.9, 23.0) | <0.001^§^ |
| Model 2 | 23.9 (23.4, 24.6) | 23.5 (22.9, 24.0) | 22.4 (21.9, 22.9) | <0.001^§^ |

Model 1 was adjusted for age (years, continuous) and menopausal status at BC diagnosis (pre-menopause, post-menopause).

Model 2 was adjusted for age (years, continuous), menopausal status at BC diagnosis (pre-menopause, post-menopause), highest education completed (middle school, high school, college education and above), ever smoked (no, yes), current alcohol use (no, yes) and family history of breast cancer (no, yes).

^*^LDL-C was estimated from Friedewald’s equation. Clinical LDL-C and LDL-C are the same biomarker but refer to different estimation methods (56).

^§^*P*-trend was signifincant at Bonferroni corrected *P*-value = 0.00147
